# Adsorptive removal of reactive yellow S3R dye from aqueous solutions using green-synthesized copper nanoparticles

**DOI:** 10.1038/s41598-025-32372-5

**Published:** 2026-01-06

**Authors:** Mohamed A. Zayed, Hossam M. Abdel-Aziz, Soha A. Abdel-Gawad

**Affiliations:** 1 Chemistry Department, Faculty of Science, Cairo University, Giza, 12613 Egypt; 2 Chemical Industries Development (CID) Company, Giza, Egypt; 3 Faculty of Postgraduate Studies for Nanotechnology, Cairo University, Giza, Egypt

**Keywords:** Ficus benjamina leaves, Green synthesis, Reactive yellow S3R removal, Zero-valent cu novel nano- particles, Wastewater treatment, Chemistry, Environmental sciences, Materials science, Nanoscience and technology

## Abstract

Industrial dye pollution poses a significant environmental threat, particularly from azo dyes like Reactive Yellow S3R (RY S3R), which are resistant to conventional treatment methods. This study investigates the efficacy of green-synthesized copper nanoparticles (CuNPs), via Ficus Benjamina leaves, for removing RY S3R from aqueous solutions. The innovative adsorbent Ficus Benjamina nano zero-valent copper (Ficus-nZVCu) was described using SEM, EDAX, and FTIR. Batch adsorption studies assessed the impact of pH, contact time, adsorbent dosage, and starting concentration on elimination efficiency. Several adsorption models were tested, and the Langmuir isotherm (q_max_ = 136.986 mg g^− 1^, R^2^ = 0.9992) revealed the best fit, suggesting monolayer adsorption. Chemisorption was indicated by the pseudo-second-order model (R^2^ = 0.9993), which was validated by the kinetic analysis. Owing to its redox activity, the green Ficus-nZVCu adsorbent is a promising material for eliminating RYS3R in light of the data acquired and the fruitful research. The impact of major operational parameters was validated using artificial neural network models and response surface methods. According to this study, green-synthesized CuNPs can effectively and safely treat dye-contaminated wastewater.

**Supplementary Information:**

The online version contains supplementary material available at 10.1038/s41598-025-32372-5.

## Introduction

Nowadays, reactive dyes are used extensively on cellulose fibres in the textile industry worldwide because of their essential characteristics^1–3^. Synthetic dyes, particularly azo dyes, lead to significant environmental contamination. These dyes are toxic, non-biodegradable, and can cause severe health hazards such as carcinogenicity and mutagenicity in aquatic organisms and humans^4,5^. Traditional wastewater treatment methods involve biological treatments^6,7^, the coagulation-flocculation methods^8,9^, electrochemical treatments^10–12^, and photocatalytic treatments^13,14^. They often fail to remove such persistent dyes effectively. Adsorption is one of the most effective technique for eliminating different pollutants and textile dyes from wastewater^15^. To facilitate regeneration and reuse in subsequent cycles, the optimal adsorbent must exhibit a high binding affinity for the target pollutant and be readily released under various desorption conditions^16^. Nanomaterials are used to create new, environmentally friendly adsorbents that enhance adsorption performance. These molecules are special owing to their small size, large surface area, and rapid reaction^17–19^.

Plant-mediated green produced nanoparticles are essential since they don’t require specific tools or harmful chemicals^20,21^. Numerous studies have recently uncovered ways for plants to produce copper nanoparticles (CuNPs) and employ them to eliminate contaminants^22^. CuNPs’ remarkable optical, electrical, catalytic, mechanical, antifungal, and antibacterial qualities have attracted much attention^23–25^. CuNPs are superior nanoparticles because they are inexpensive, widely available, have a large surface area, produce better yields, and react more quickly under mild reaction conditions than the traditional adsorbent^26^. Thus, this is the primary motivation behind working on CuNPs.

**Fig. 1:**
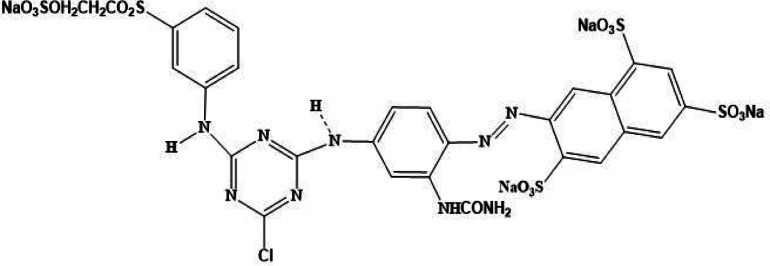
Molecular structure of reactive yellow S3R dye.

Reactive yellow S3R (RYS3R) dye, (C_28_H_20_ClN_9_Na_4_O_16_S_5_), is mainly used in the textile industry to dye cellulose fiber, cotton, and polyester (Fig. 1). Given that exposure to the dye can result in several adverse health effects, including acute bronchitis, skin irritations, mutations, and bladder cancer, the liberation of S3R into the environment constituted a serious risk to public health^27,28^. Consequently, wastewaters containing RYS3R need to be treated before released into the environment. Given its high molecular weight, this dye’s limited adsorption and low degradability on various sorbents make it particularly interesting. Because of its refractoriness and few adsorption investigations, the RYS3R was selected to be studied in this work.

This study aims to assess the potential of green-synthesized CuNPs for efficiently removing Reactive Yellow S3R from contaminated water. FT-IR and SEM examined the green synthesis of zero-valent copper nanoparticles via Ficus Benjamina leaf extracts. To reach the optimal dye removal conditions, the authors investigated the impacts of the working parameters on the dye removal process, as well as different isotherms used to fit the collected data. This eco-compatible approach contributes to sustainable nanotechnology and provides an effective solution for the remediation of textile dyes.

## Materials and methods

### Materials

Textile dyes Reactive yellow S3R (C_28_H_20_ClN_9_Na_4_O_16_S_5;_ Molecular Weight: 1026.24) were acquired from local textile industry waste and used without further purification. Copper sulfate (CuSO_4_.5H_2_O, Sigma-Aldrich Co. Ltd.), Ficus leaves (Ficus tree, Egypt), and reagent grade were all of the highest purity. A digital pH meter with a microprocessor was utilized to measure the solution pH value (Jenway, UK). To balance the pH, changes to the solution were made using solutions of HCl and NaOH.

### Ficus copper nanoparticles preparation (Ficus-nZVCu)

**Fig. 2:**
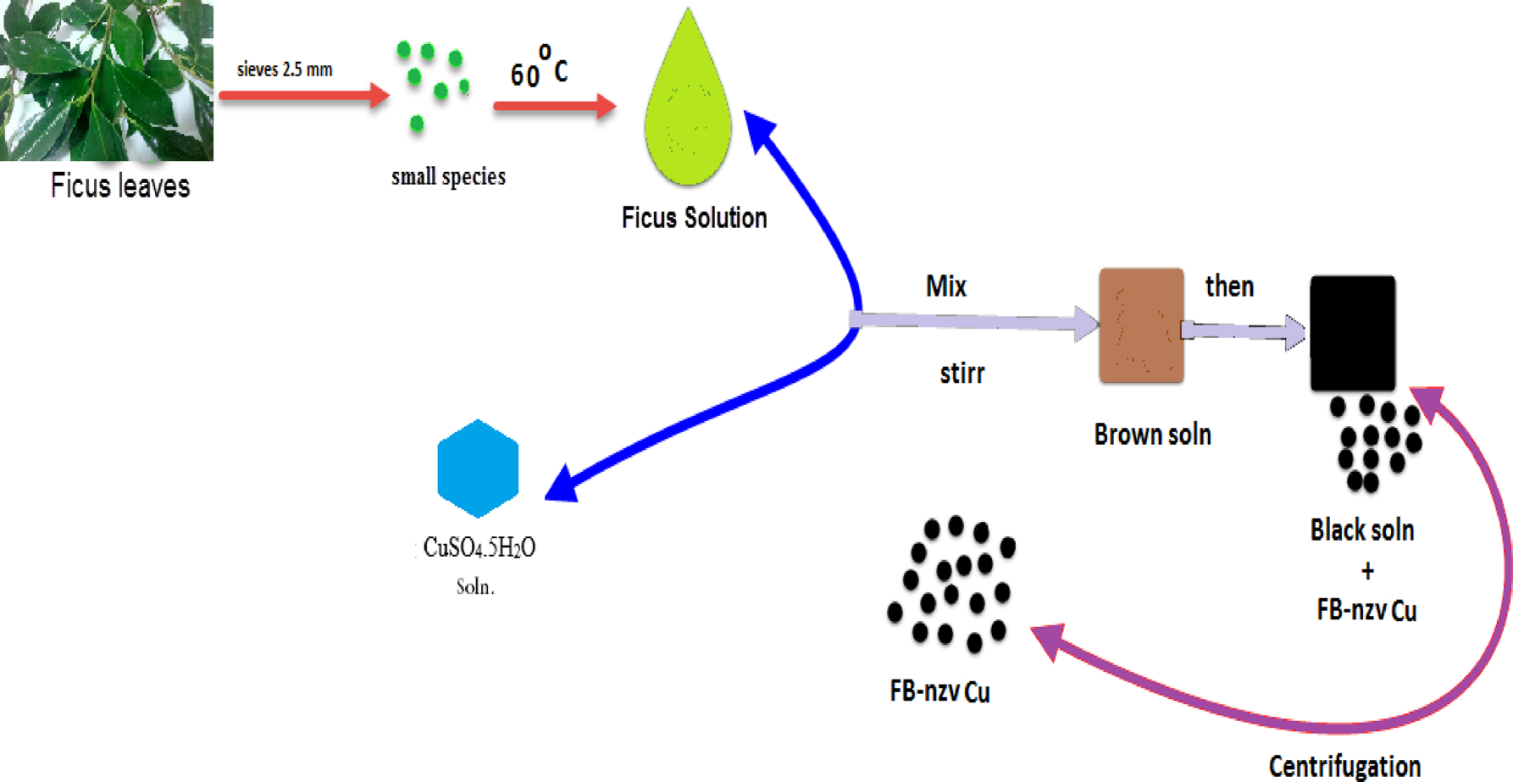
Methodology of Ficus nanoscale-ZVCu particle preparation.

Ficus Benjamina leaves were washed, dried in an oven at 105 °C, subdued into small minutes and sieved through the sieve 2.0 mm. Plant extract of 4 g of leaves added to 50 mL of distilled water, heated at 80 °C for 60 min, then filtered. A copper sulfate solution was prepared by dissolving 0.75 g of CuSO_4_.5H_2_O in 25 mL of distilled water. To synthesize Ficus-nZVCu particles, 50 mL of Ficus leaf extract was added dropwise to 100 mL of copper solution and stirred for 30 min to produce nanoscale particles. The color of the Ficus extract in the reaction container had altered from yellowish to brown, then to black, indicative of the formation of Ficus-nZVCu particles^29^. Ficus-nZVCu nanoparticles were isolated after centrifuging for 5 min. The precipitate was rinsed with 95% ethanol. Ficus-nZVCu nanoparticles were dried at 65 °C and stowed in a desiccator^30^. The preparation for the green Ficus-nZVCu nanoparticles is presented in Fig. 2.

### Characterization of Ficus-nZVCu

Observation of the morphology of the Ficus-ZVCu by SEM. Ficus-ZVCu nanoscale particles coated with gold are scanned to provide high contrast, allowing for accurate measurement of particle size. EDAX was utilized to ascertain the composition of the sample. FTIR is accomplished in 400–4000 cm^−1^ range to detect the vibrational groups present. These techniques help to identify surface functional groups and confirm the reduction of copper ions.

### Batch technique

A stock solution of 100 mg L^− 1^ of RYS3R was diluted to the desired concentration. In each experiment, a different dye solution with a known concentration was mixed with varying doses of Ficus-nZVCu nanoparticles. The percentages of removal were calculated utilizing the mathematical Eq. (1) based on the spectra of dye solutions at λ_max_ = 416 nm for RYS3R.

1 $${\text{Sorption }}\left[ \% \right] = \left[ {{{\left[ {C_{{\mathbf{0}}} - C_{{\mathbf{e}}} } \right]} \mathord{\left/ {\vphantom {{\left[ {C_{{\mathbf{0}}} - C_{{\mathbf{e}}} } \right]} {\left[ {C_{{\mathbf{0}}} } \right]}}} \right. \kern-\nulldelimiterspace} {\left[ {C_{{\mathbf{0}}} } \right]}}} \right] * {\text{1}}00$$

Where C_0_ and Ce represent the initial and equilibrium concentrations of the dyes (mg L^**− 1**^). The removal capacity at equilibrium was attained by using Eq. (2):

2 $$q_{{\text{e}}} = {{\left[ {\left[ {C_{{\mathbf{0}}} - C_{{\mathbf{e}}} } \right]V} \right]} \mathord{\left/ {\vphantom {{\left[ {\left[ {C_{{\mathbf{0}}} - C_{{\mathbf{e}}} } \right]V} \right]} m}} \right. \kern-\nulldelimiterspace} m}$$

Where V is the volume of aqueous solution (L), q_**e**_ is the equilibrium removal capacity (mg g^− 1^), and m is the wt. of the Ficus-nZVCu nanoparticles (g)^31,32^.

### Adsorption equilibrium models

 The adsorption equilibrium models are crucial for calculating and evaluating the various possible interactions between the RYS3R molecules and the adsorbent that occur during adsorption. These suggested models involved the linear Dubinin-Radushkevich, Elovich, Flory–Huggins, Fowler, Freundlich, Halsey, Harkins-Jura, Henry, Hill-Deboer, Jovanovic, Langmuir, Redlich-Peterson, Sips, Temkin, and Toth isotherm models, respectively. The equations used for the different models in the calculations of various adsorption parameters are depicted in Table S1.

### Kinetic equilibrium models

 Kinetic equilibrium isotherm models are essential for investigating the mechanism of adsorption and the reaction processes that occur in RYS3R removal processes, using different possible reaction orders, including Pseudo-first-order, Pseudo-second-order, Elovich, Fowler, Intra-Particle Diffusion, Avrami, Bangham, Boyd, and liquid film diffusion linear isotherm models. The equations used in different kinetic equilibrium models for studying adsorption processes in this text are shown in Table S2.

### Thermodynamic study

To explore how temperature affects the removal of RYS3R dyes by Ficus-nZVCu materials, thermodynamic calculations were done to quantify the degree of spontaneity. The experiment was done at different temperatures to examine the thermodynamics of the adsorption reaction. The Gibbs free energy (∆G), entropy (∆S), and enthalpy (∆H) were determined^20,33^.

3 $$\Delta {\text{G}} = - {\text{RT}}\;{\text{ln}}\;{\text{K}}_{{\mathbf{d}}}$$

4 $${\text{K}}_{{\mathbf{d}}} = {{{\text{q}}_{{\mathbf{e}}} } \mathord{\left/ {\vphantom {{{\text{q}}_{{\mathbf{e}}} } {{\text{C}}_{{\mathbf{e}}} }}} \right. \kern-\nulldelimiterspace} {{\text{C}}_{{\mathbf{e}}} }}$$

5 $$\Delta {\text{G}} = \Delta {\text{H}} - {\text{T}}\Delta {\text{S}}$$

6 $${\text{Ln}}\;{\text{K}}_{{\mathbf{d}}} = \left( {{{\Delta {\text{S}}} \mathord{\left/ {\vphantom {{\Delta {\text{S}}} {\text{R}}}} \right. \kern-\nulldelimiterspace} {\text{R}}}} \right) - \left( {{{\Delta {\text{H}}} \mathord{\left/ {\vphantom {{\Delta {\text{H}}} {{\text{RT}}}}} \right. \kern-\nulldelimiterspace} {{\text{RT}}}}} \right)$$

Where R (universal gas constant) and K_**d**_ (adsorption equilibrium constant). ∆S and ∆H were computed using the slope and intercept of 1/T versus Ln K_**d**_, respectively.

### Linear modeling algorithms for statistical data analysis

Linear regression analysis, SPSS 24, was used to study the influences of several parameters, including solution pH, contact time, particle weight, stirring rate, temperature, and different concentrations of dyes. Total squares and the impact of the entire model were displayed by the ANOVA program^34,35^.

#### Response surface methodologies (RSM)

The impact of different operational factors on the batch was studied. If the* P*-value is a significant factor for the removal procedure (significant ˂ 0.05 ˃ isn’t significant). Deduction of RYS3R dye in the adsorption process can be assumed to follow Eq. (7):

7 $${\text{R\% = Bo + B1}}{\text{.X1 + B2}}{\text{.X2 + B3}}{\text{.X3 + B4}}{\text{.X4 + B5}}{\text{.X5}}$$

Where R is the removal percent, B° (constant), X_1_ (pH), X_2_ (time), X_3_ (dose), X_4_ (stirring rate), X_5_ (concentration), and X_6_ (temperature).

#### Artificial neural network (ANN)

An artificial neural network with input, hidden, and output layers was developed using a multilayer perceptron and “Multilayer Perceptron Backpropagation (MLPB)” to predict the efficiencies of wastewater contaminants removal. The input layer received data from six experimental factors (dose, pH, time, stirring rate, initial concentration, and temperature), and the hidden layer contained different neurons. ANN models using a multilayer perceptron (backpropagation method) provide the relationship between different values, as well as the importance and normalized importance of each effect^36^.

### Reusability of Ficus-nZVCu

To meet both ecological and economic objectives and determine the cost-effective application of the adsorbent, its reusability is essential. With a concentration of 50 mg L^− 1^, an adsorbent dosage of 0.2 g L^− 1^, a pH of 6, and a contact time of 60 min, Ficus-nZVCu was used to remove the RYS3R dye. Subjecting a reacted Ficus-nZVCu to a fresh dye solution, the studies were conducted up to five times to examine its reusability further. Following each reaction, the Ficus- nZVCu was quickly removed from the solution, centrifuged for ten minutes, and then cleaned with ethanol. It was dried at 45 °C in an oven before being used for subsequent adsorption recycling.

## Results and discussion

### Characterization of Ficus-nZVCu

Different analytical and morphological tools were used to characterize and analyze the Ficus-nZVCu nano adsorbent.

#### SEM and EDAX

**Fig. 3:**
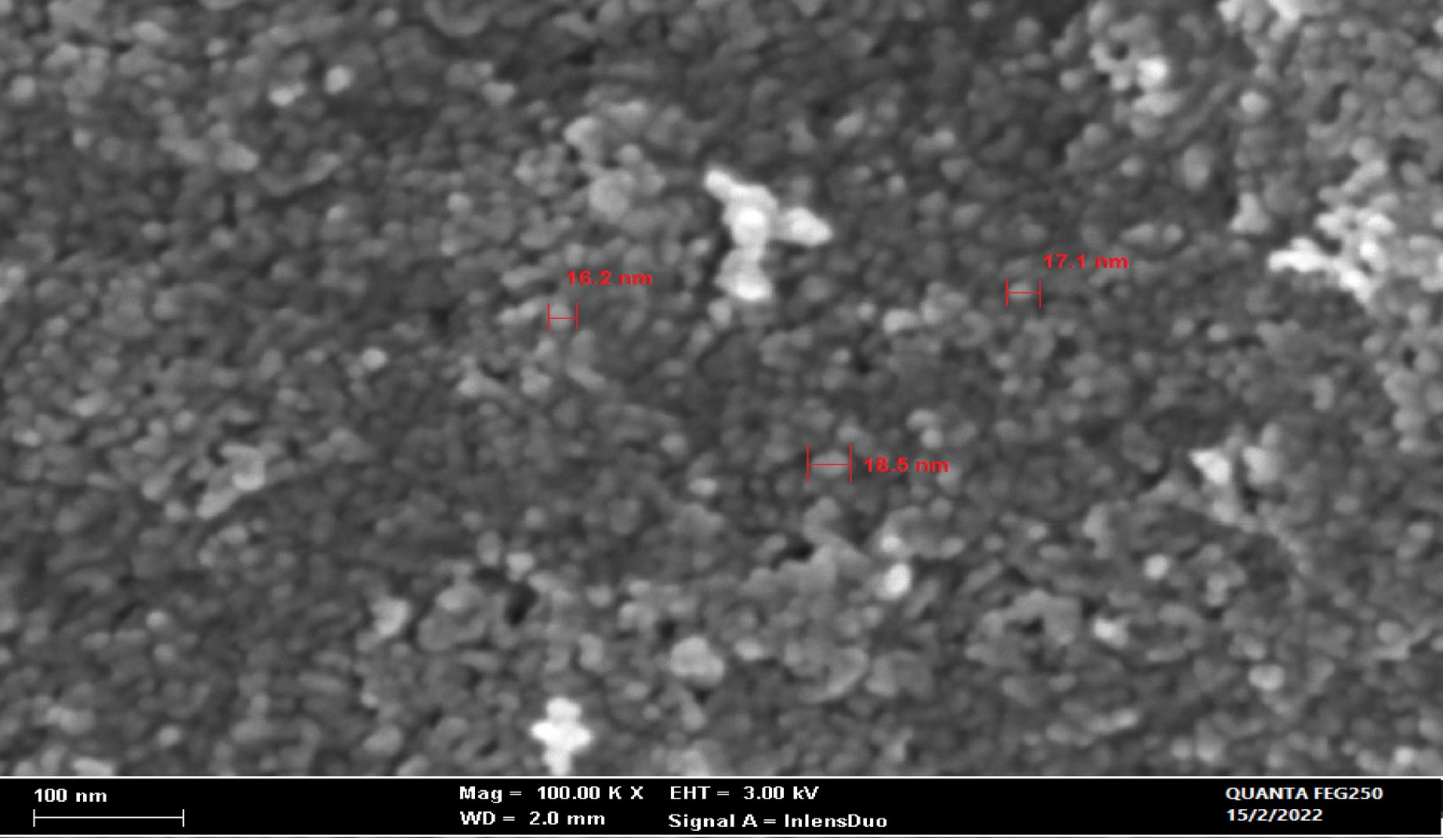
Ficus-nZVCu samples SEM.

SEM result of the morphological test of the prepared Ficus-nZVCu is presented in Fig. 3. The SEM image depicts semi-spherical nanoscale particles with sizes varying from 16.2 to 18.5 nm. In some cases, nano agglomerations are detected and linked to the sample centrifugation procedure. Several pores are also involved in the spread and transportation of organic dye to the inner Ficus-nZVCu.

**Fig. 4:**
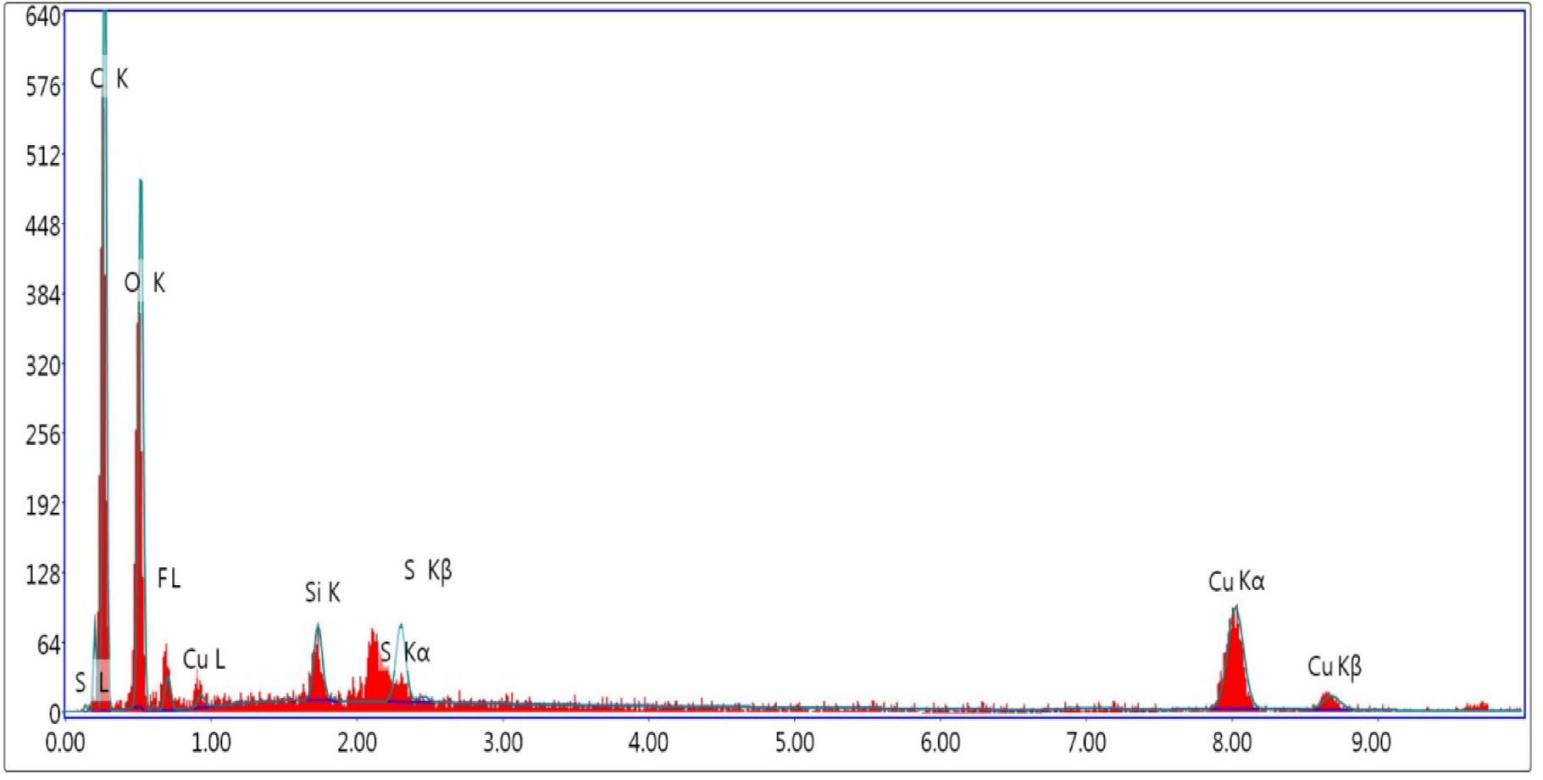
EDAX of the prepared Ficus-nZVCu sample.

The EDAX result is depicted in Fig. 4, confirming the successful synthesis of Ficus-nZVCu particles. The occurrence of Cu peak points to nano zero-valent particles. Other peaks include Ficus extract S, Si, O, and C^17^.

#### FT-IR measurements

**Fig. 5:**
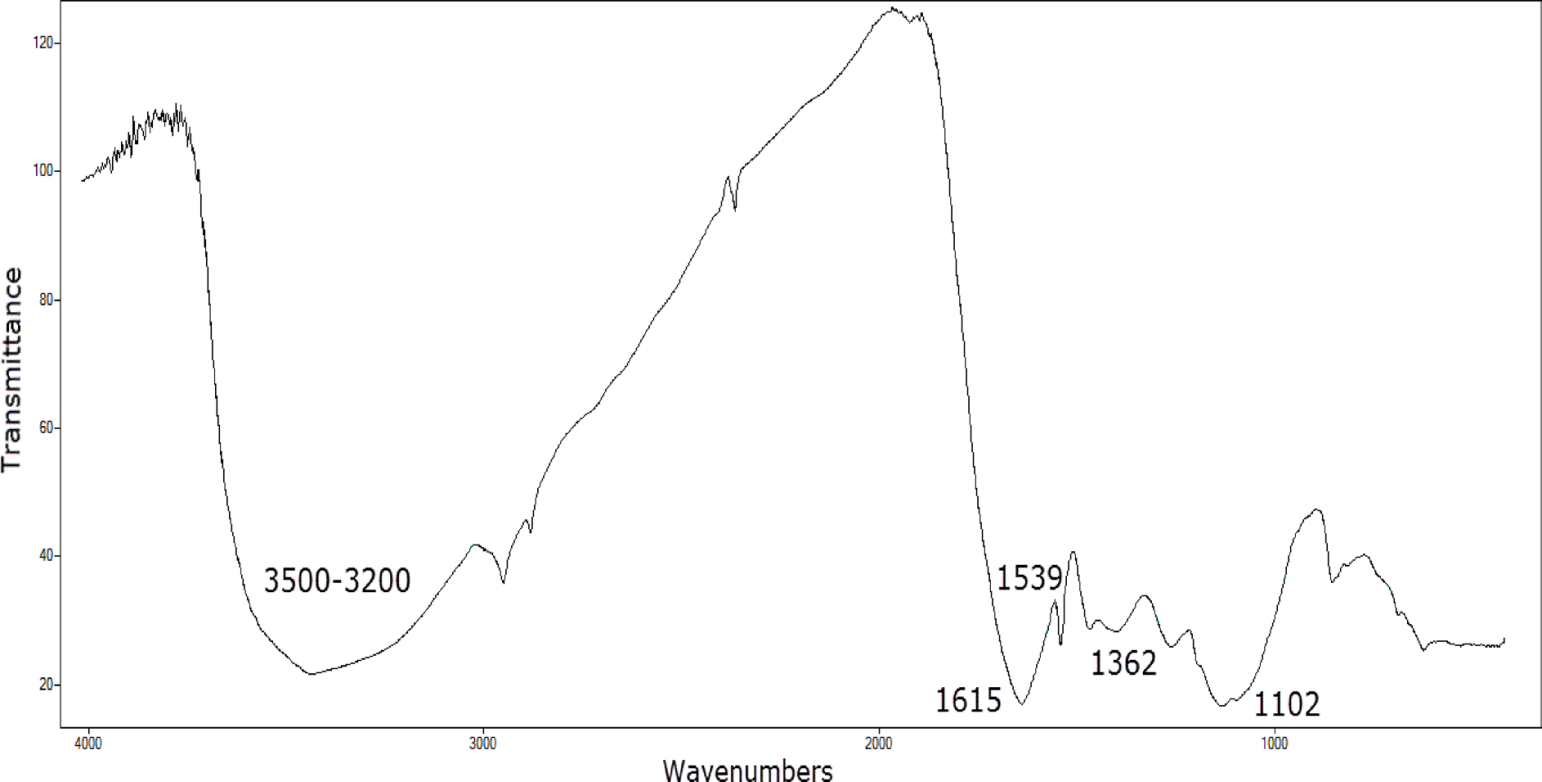
FT-IR spectrum of Ficus-nZVCu.

The FT-IR measurements were used to detect polar groups in the Ficus-nZVCu adsorbent that were involved in and affected the removal process. The obtained results are presented in Fig. 5. Polyphenol’s broadband peak, which appeared at 3400 to 3100 cm^− 1^, is related to OH stretching vibration^37,38^, whereas the C = O band appeared at 1615 cm^− 1^^39^. The Ficus amide peak is detected at 1520 cm^− 1^^40^, and the polyphenolic aromatic ring C = C stretching vibration is found at 1380 cm^− 1^^41^. Figure 5 refers to the presence and the strength of phenolic compound peaks capable of reducing Cu and serving as an indicator of synthesized Ficus-nZVCu^17^.

#### Spectrum of dyes

The absorption spectra of RYS3R dyes 50 mg L^**− 1**^ were determined in the 190–800 nm wavelength range. Before the reaction, the spectrum shows the major peaks of RYS3R dye at 416 nm.

### Effect of working considerations

#### Dye solution pH influence

**Fig. 6:**
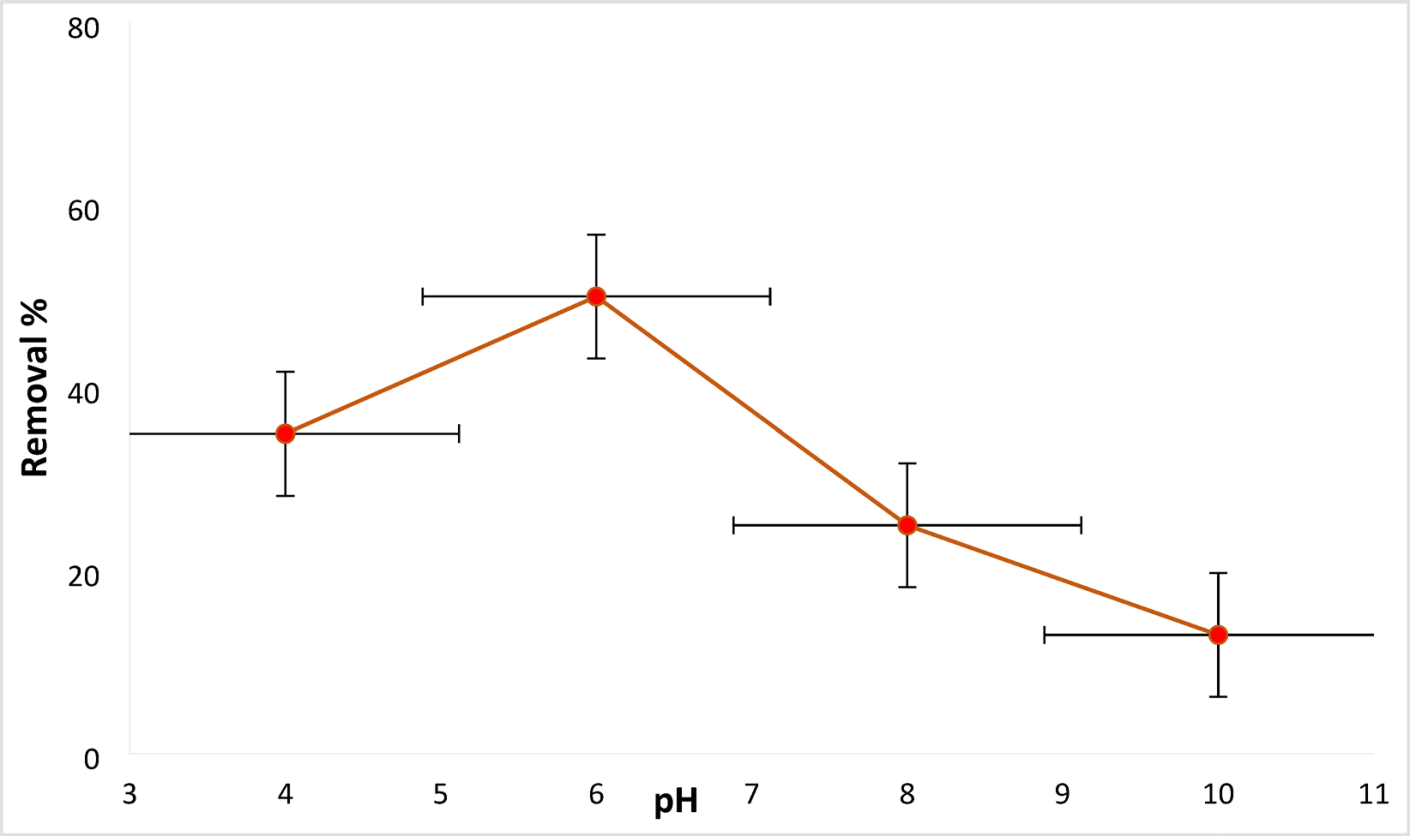
Effect of solution pH on the removal of the RYS3R dye.

The effect of solution pH on the removal of dyes by the tested adsorbent was examined, and the outcomes are presented in Fig. 6. The dye solution pH plays a major significant part in the dye removal efficiency procedure. RYS3R dyes were studied at different solution pH ranging from 4 to 10 under selected optimal circumstances (dose 0.2 g L^− 1^, concentration 50 mg L^− 1^, time 60 min, stirring 100 rpm). The removal varied with the variation in solution pH for RYS3R (35, 50, 25, and 13%), as depicted in Fig. 6. Ficus zero valent copper has a low zero charge point 5.7^24^. The attraction between the (positively charged Ficus-nZVCu surface and negatively charged RYS3R dyes) was amplified at solution pH 6. At dye solution pH 8 < pH_pzc,_ the surface is charged negatively, and repulsion occurs with the dye particles (pH 10: precipitated part of the dose), resulting in low removal efficiency. Therefore, a dye solution with a pH of 6 is considered optimal for removing RYS3R dyes^42^.

#### Dye solution contact time influence

**Fig. 7:**
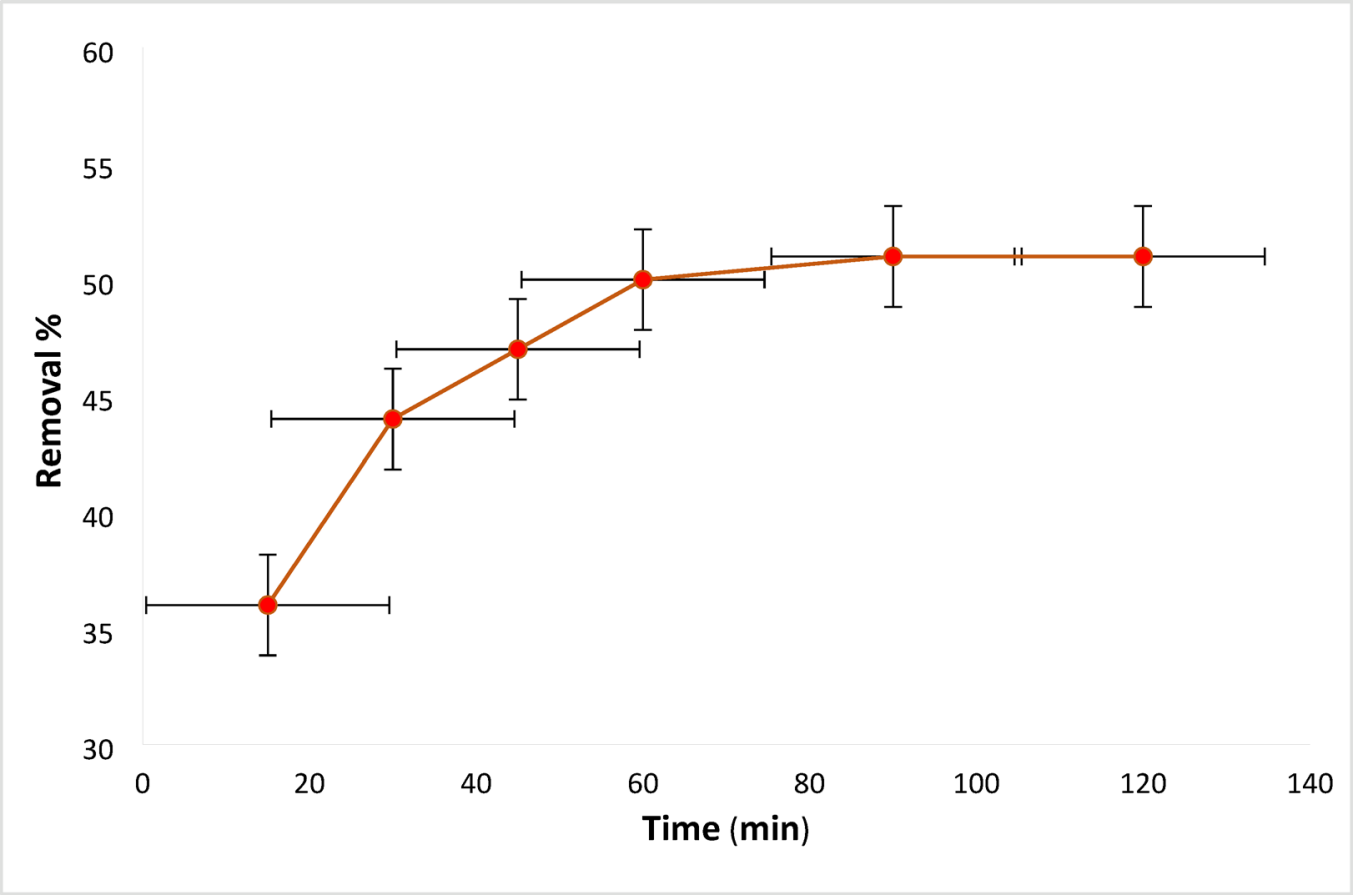
Effect of contact time on the removal of the RYS3R dye.

Figure 7 shows the outcomes of the adsorption process, which depends critically on the contact duration between dyes and adsorbents. Contact time’s effect on removal was examined at 15, 30, 45, 60, 90, and 120 min. intervals. Using 0.2 g L^**−** 1^ of Ficus-nZVCu at solution pH 6 and a stirring rate 100 rpm, the percent of the dye removal for RYS3R dye concentrations of 50 mg L^− 1^ is found to be 36, 44, 47, 50, 51, and 51%, as shown in Fig. 7. The amount of electrically attracted molecules between the positively charged Ficus-nZVCu surface and the negatively charged dyes increased with the increase in contact time, which gradually increased the concentration of the contaminant in the empty sites of the nanoparticles. Consequently, the removal reaches a maximum and is nearly constant. At a contact time of 60 min, the fit proportion of dye removal occurs.

#### Ficus-nZVCu adsorbent dose influence

**Fig. 8:**
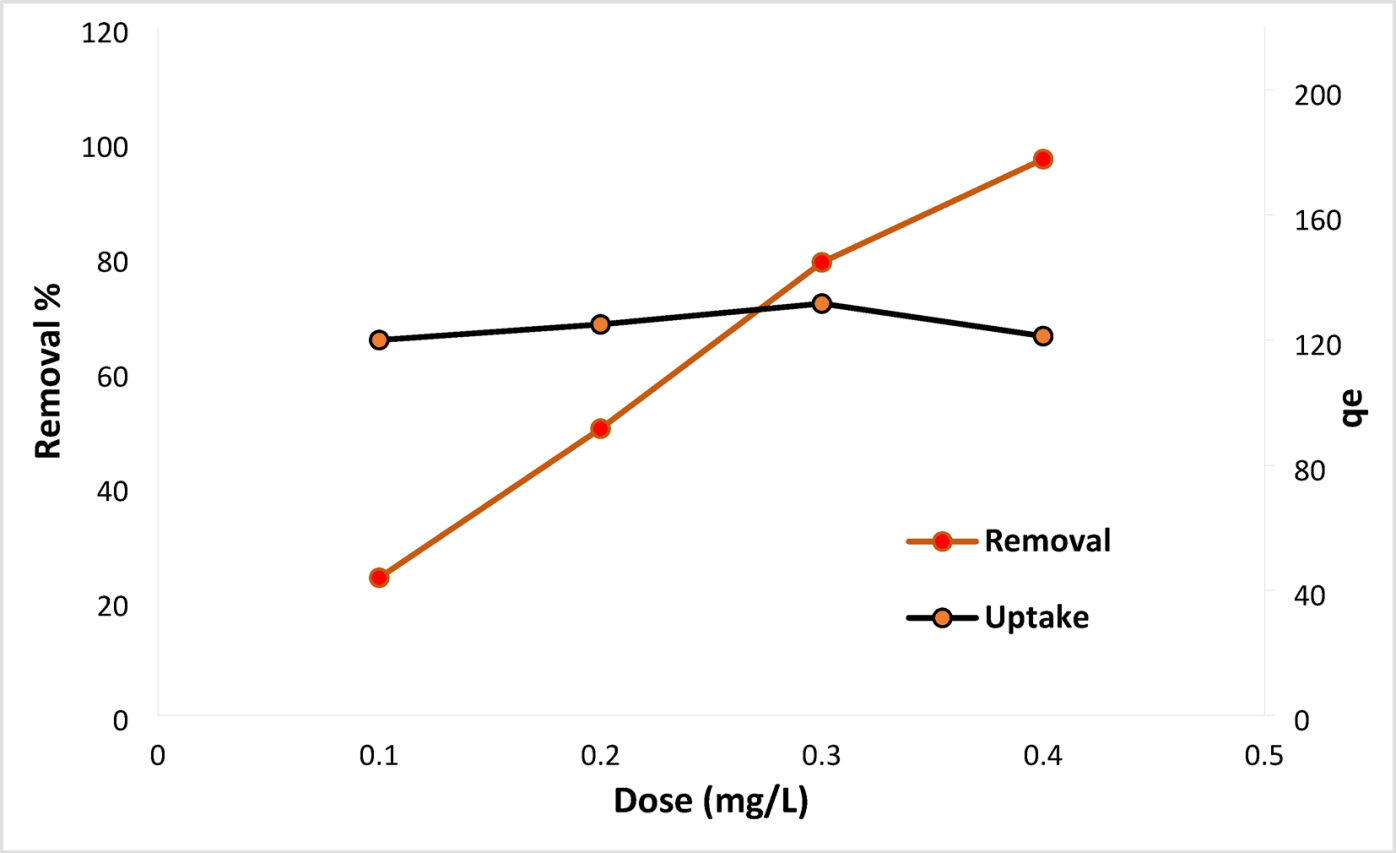
The optimum effective dose and uptake on the RYS3R dye removal.

Figure 8 displays the outcomes of an experiment on the adsorbent dosage effect of RYS3R dye on the removal process. The efficiency of RYS3R dye removal was investigated when the doses of RYS3R dye were adjusted from 0.1 to 0.4 g L^− 1^ while keeping the rest of the functional conditions (solution pH initial for RYS3R = 6, time 60 min, stirring rate 100 rpm, and concentration 50 mg L^− 1^). As a result, the RYS3R removal percentages at altered doses are found to be (24, 50, 79, and 97%). The optimal dosage for eliminating RYS3R dyes was found to be approximately 0.3 g L^− 1^. Therefore, the higher Ficus-nZVCu dose raises the number of vacant sites, which increases the RYS3R dye removal percentage.

#### Stirring rate on RYS3R dye removal influence

**Fig. 9:**
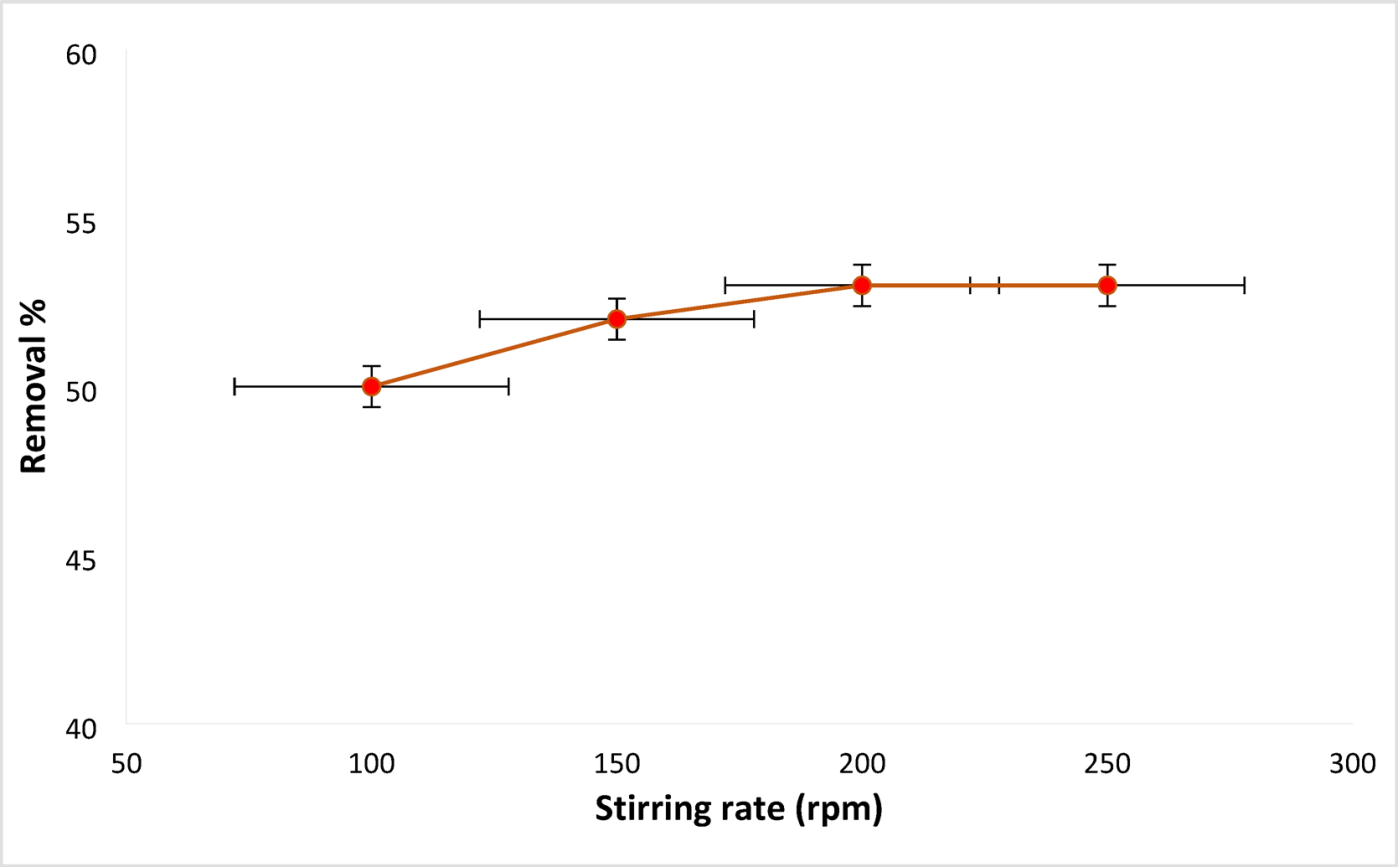
Effect of stirring rate on the removal of the RYS3R dye.

Figure 9 demonstrates RYS3R dye removal by Ficus-nZVCu at a various stirring rate of 100 to 250 rpm at solution pH 6, time 60 min, and a RYS3R dye concentration of 50 mg L^− 1^. The removal of RYS3R dye concentrations under these conditions is found to be 50, 52, 53, and 53%. Therefore, the selected optimal stirring rate for the removal of dyes is 100 rpm.

#### RYS3R concentration influence

**Fig. 10:**
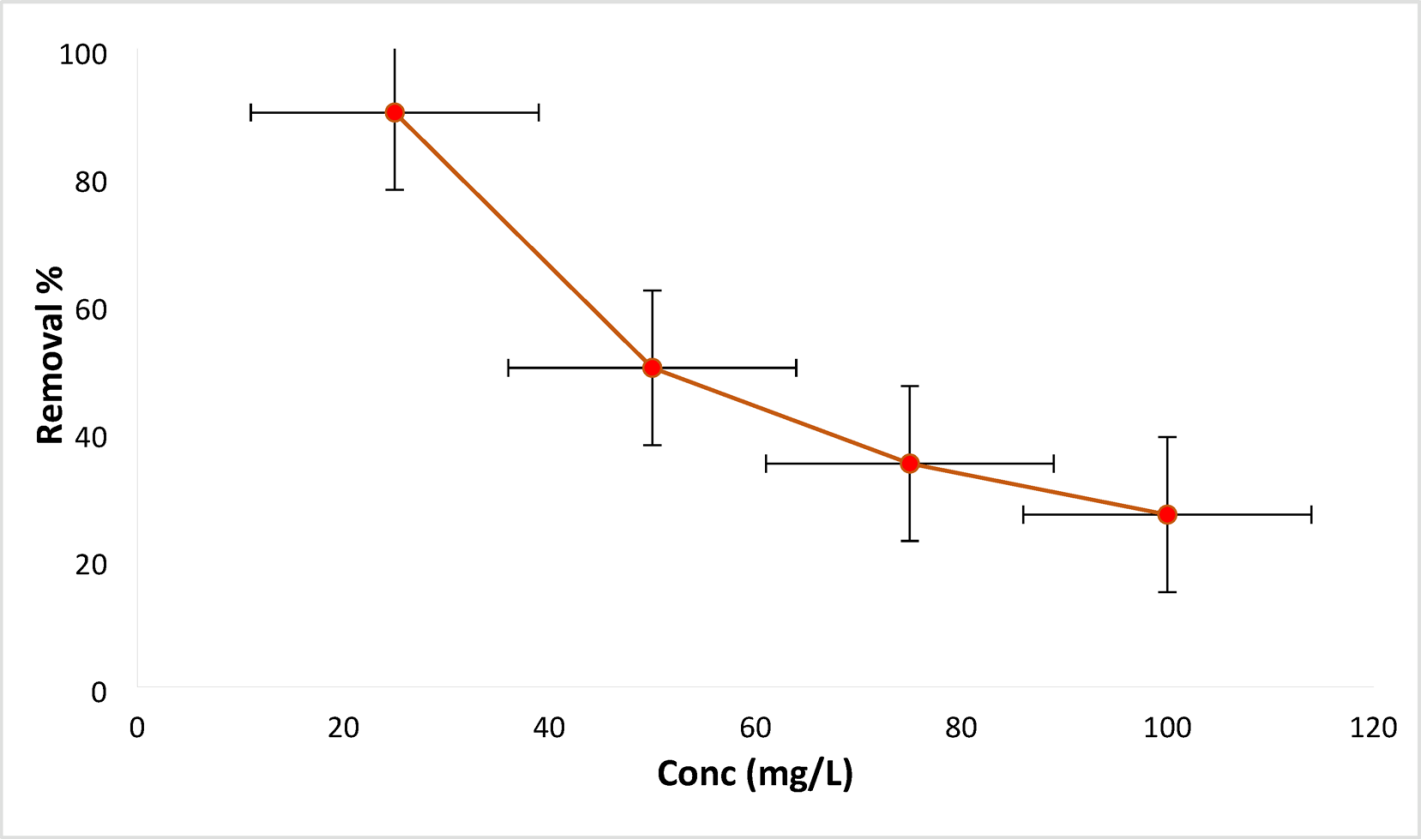
Effect of concentration on the removal of the RYS3R dye.

The influence of 0.2 g L^− 1^ of Ficus-nZVCu particles on the removal of RYS3R dye solution with different concentrations of 25, 50, 75, and 100 mg L^− 1^ at solution pH 6 for 60 min. The removal efficiencies for RYS3R dyes are found to be (90, 50, 35, and 27%), depicted in Fig. 10. The removal ratio is high due to the abundance of empty adsorption sites at low concentrations at the start of the study; however, as RYS3R dye solution concentration increases gradually, this ratio decreases owing to site saturation.

#### RYS3R temperature influence

**Fig. 11:**
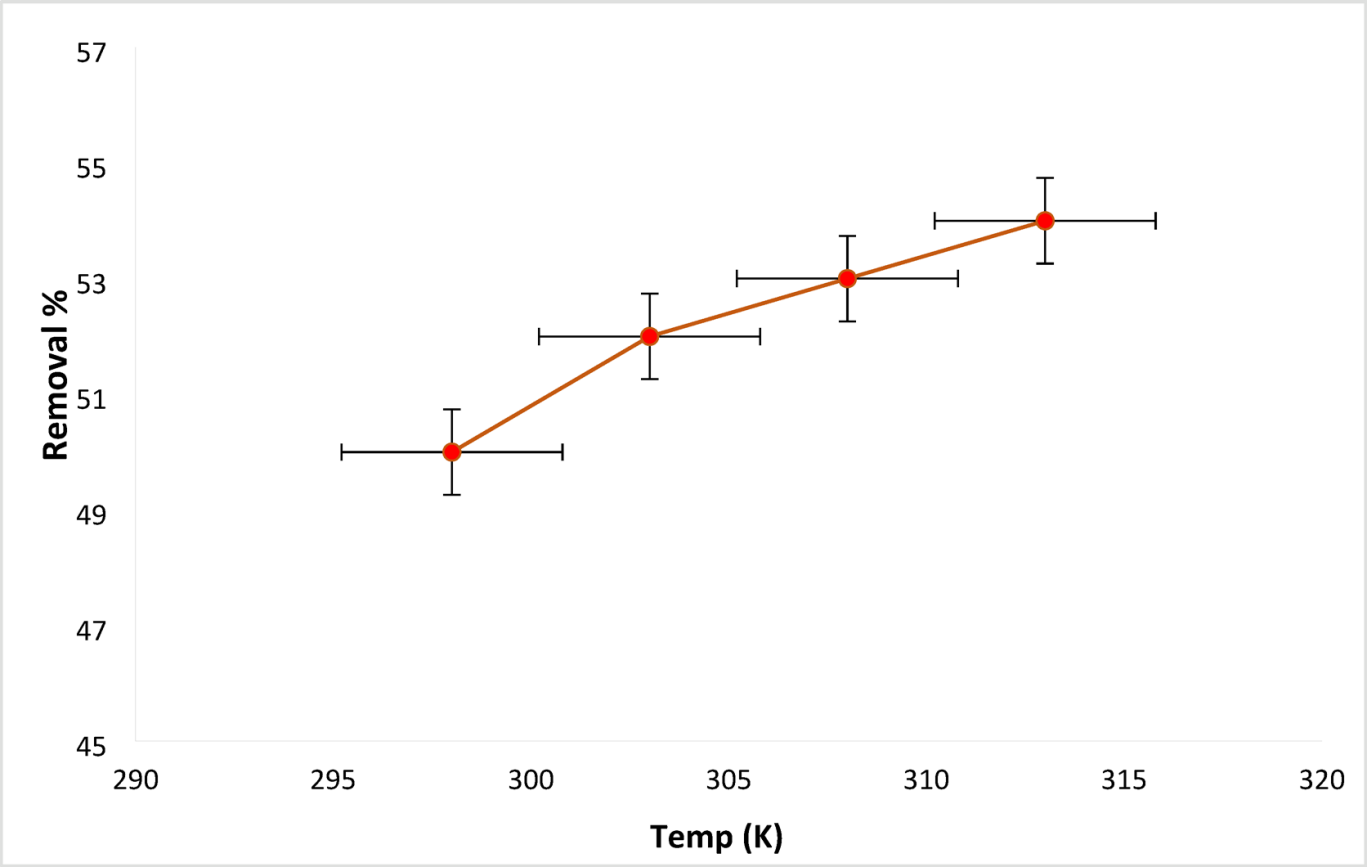
Effect of temperature on the removal of the RYS3R dye.

The effect of temperature on the rate of RYS3R dye removal from adsorption onto Ficus-nZVCu particles was examined at various temperatures: 298, 303, 308, and 313 K, with a solution pH of 6, contact time of 60 min, a concentration of 50 mg L^− 1^, and an adsorbent dose of 0.2 g L^**−** 1^. The removal efficiencies are found to be for RYS3R 50, 52, 53, and 54%, as revealed in Fig. 11. As predicted, raising the temperature slightly increases the removal efficiency, which means the adsorption of dyes on Ficus-nZVCu particles is, to some extent, endothermic. The reason for increasing removal efficiency with increasing temperature is probably due to the strong adsorptive powers between the molecules of the adsorbed phases as well as between the RYS3R dye molecules and the Ficus-nZVCu active sites (chemical adsorption). Additionally, in some circumstances, the temperature increase affects the solubility of the dye molecules, which consequently has a substantial impact on the removal efficiency^43,44^.

### Adsorption isotherm investigations

#### Linear isotherm models

**Fig. 12:**
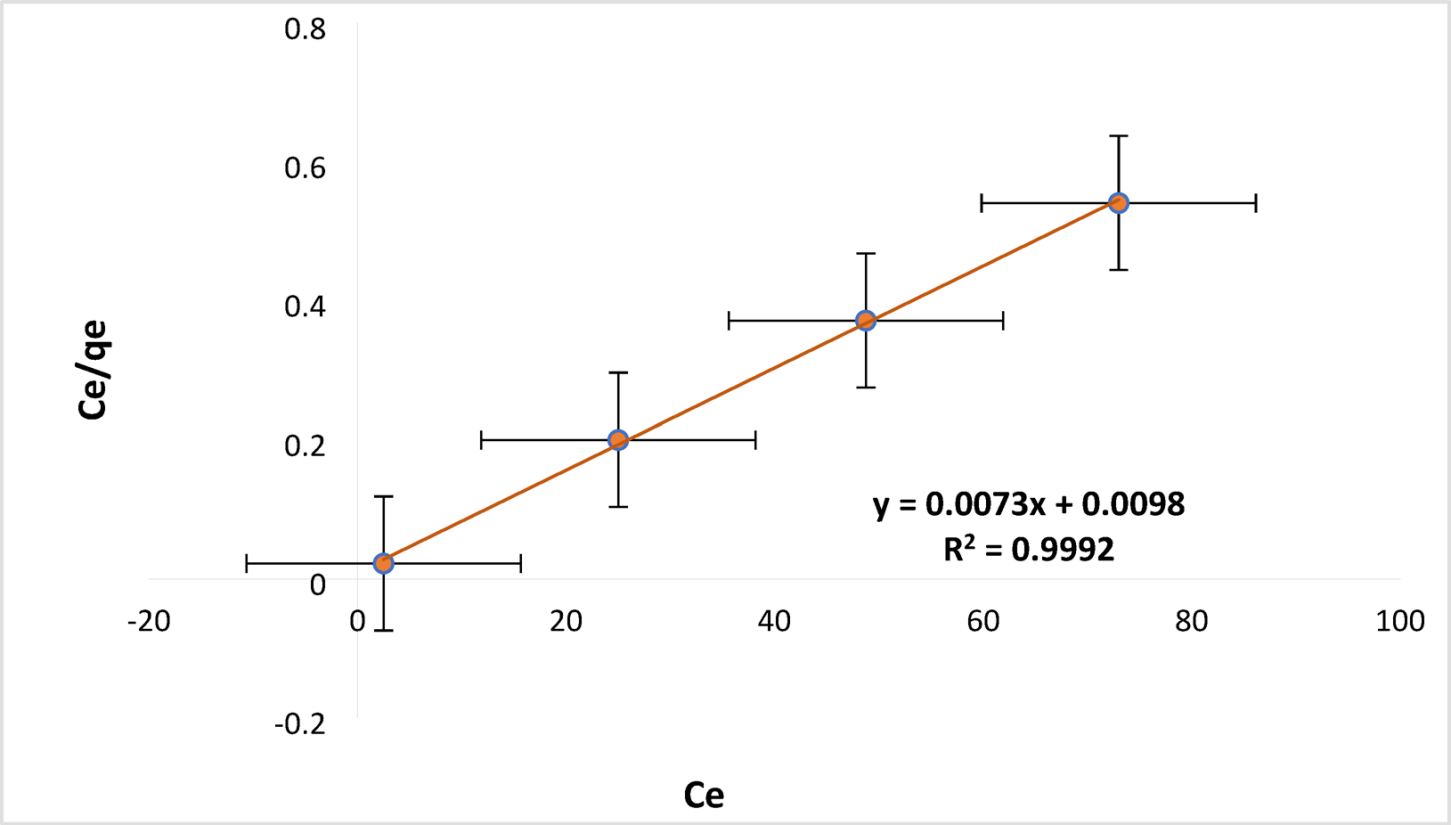
Langmuir isotherm studies for linear adsorption models [solution pH 6, dose 0.2 mg L^− 1^, time 60 min, stirring 100 rpm, concentration (25, 50, 75, and 100 mg L^− 1^)].

 The outcomes of the study of the models of adsorption isotherm are shown in Table S3, and represented graphically in Figure S1. The Langmuir q_e_ value (cal.) = 136.986 is approximately same to q_e_ (exp.) = 135 of the study for RYS3R. According to the Table S3, the Langmuir model for RYS3R dye fits the isotherm well, with higher correlation coefficients (R^2^ = 0.9992) than other linear adsorption isotherm models and a higher maximum adsorption capability (q_max_) of 136.986 mg g^−1^, as illustrated in Fig. 12.

#### Non-linear isotherm models

**Fig. 13:**
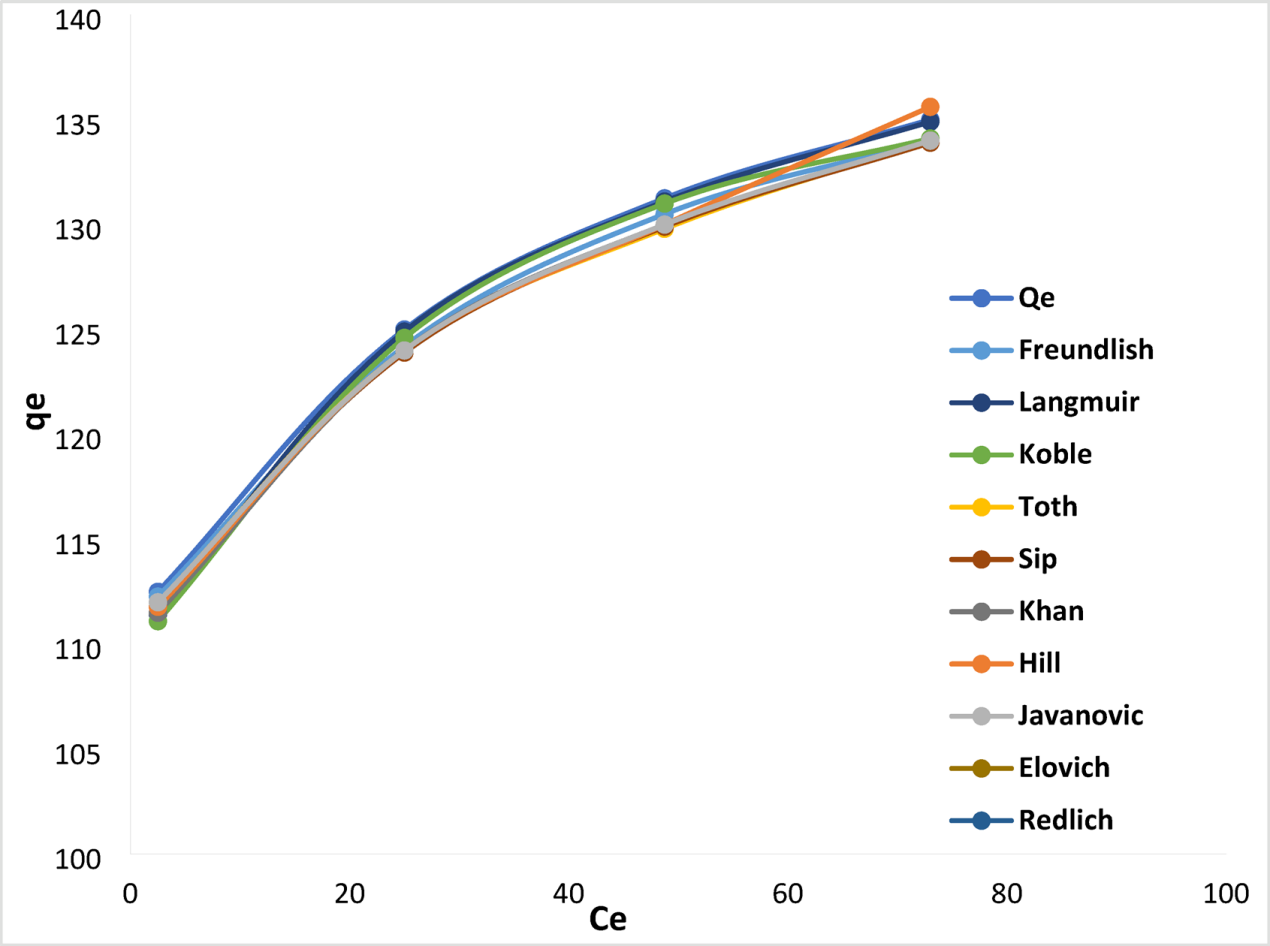
Isotherm studies for non-linear adsorption models [solution pH 6, dose 0.2 mg L^− 1^, time 60 min, stirring 100 rpm, concentration (25, 50, 75, and 100 mg L^− 1^)].

According to Table 1; Fig. 13, the findings collected showed that the Langmuir model fit the isotherm well, with a low error sum (error sum = 2.645) when compared to other non-linear adsorption isotherm models.

**Table 1:** Parameters for different non-linear isotherm models.

	Isotherm model	Error sum
		RYS3R
1.	Freundlich	5.089
2.	Elovich	6.899
3.	Hill	7.105
4.	Jovanovic	7.651
5.	Khan	8.919
6.	Kolbe	6.018
7.	Langmuir	2.645
8.	Redlich	4.827
9.	Sips	8.644
10.	Troth	8.401

### Kinetics studies

#### Linear kinetics studies

**Fig. 14:**
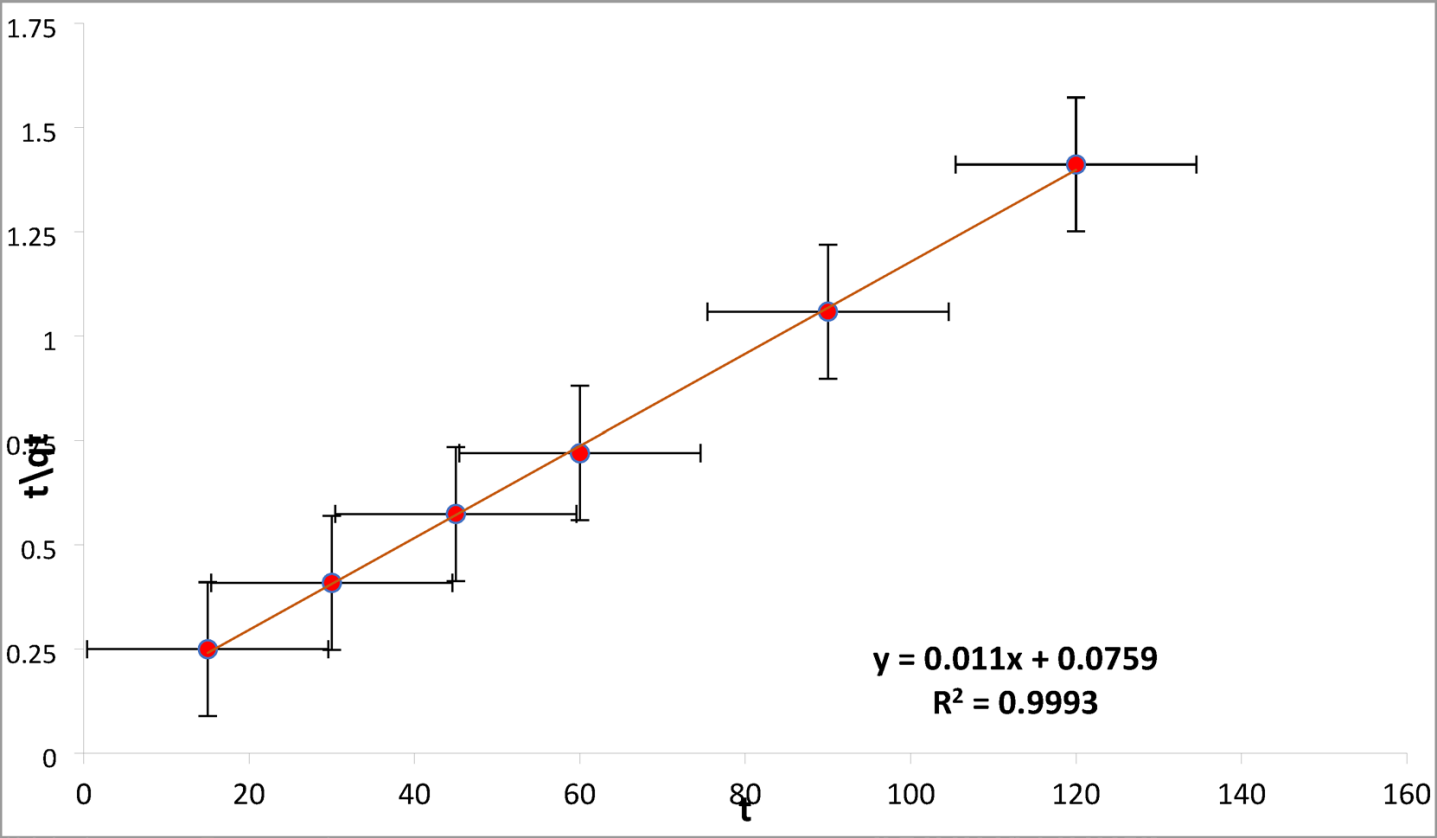
Pseudo-second order kinetic studies for linear models, [pH 6, dose 0.2 mg L^− 1^, time (15, 30, 45, 60, 90, 120 min), stirring 100 rpm, concentration 50 mg L^− 1^].

 The kinetics data obtained by applying different kinetic isotherm models is shown in Table S4, and graphically represented in Figure S2. The PSO qe value (cal.) = 90.909 is approximately same to qe (exp.) = 85 of the study for RYS3R. According to the Table S4, the PSO model for RYS3R dye provides a better fit to the data than other kinetic isotherm models, with excellent correlation (R² = 0.9993), as illustrated in Fig. 14.

#### Non-linear kinetics studies

**Fig. 15:**
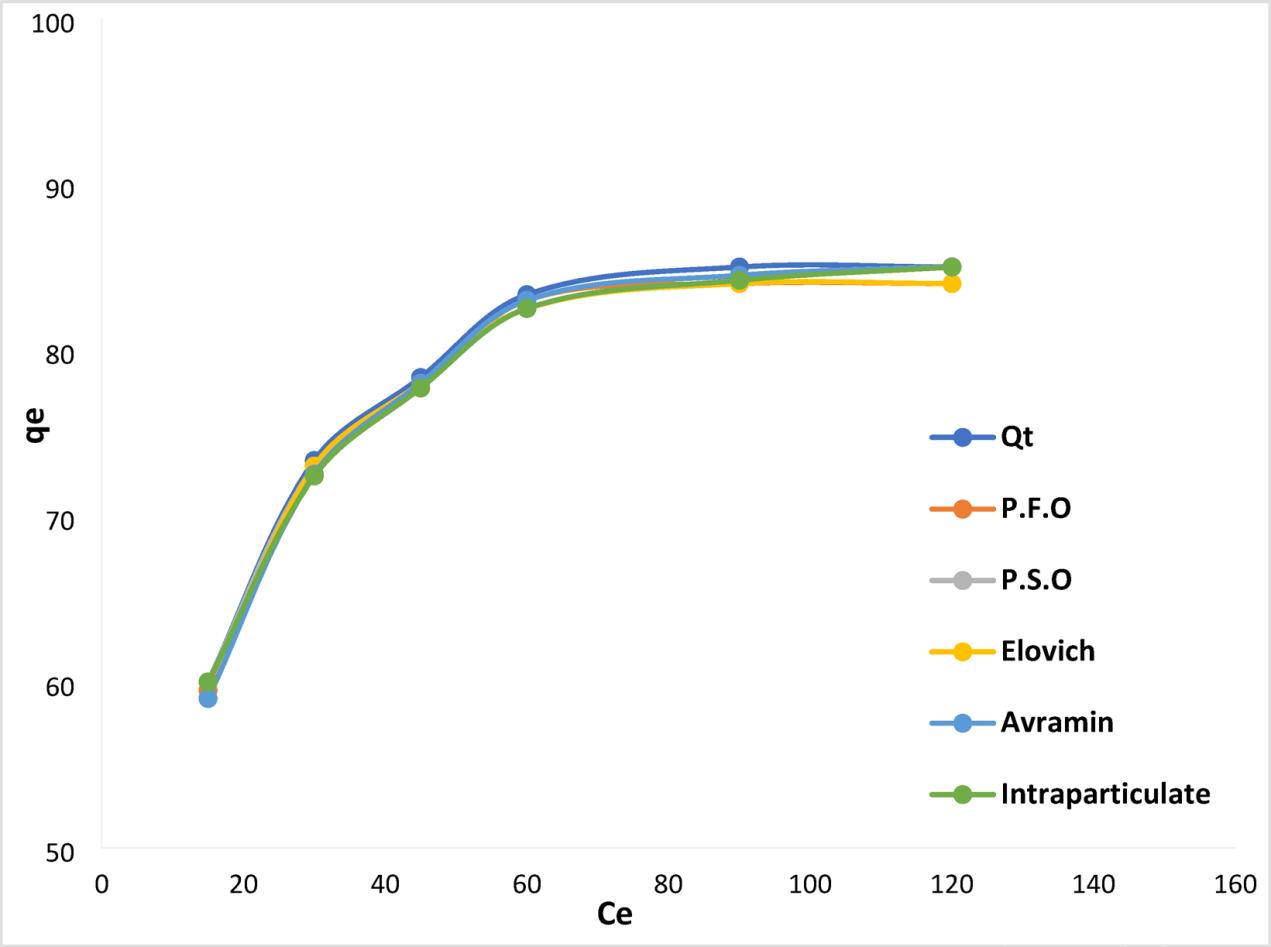
Kinetic studies for non-linear models [pH 6, dose 0.2 mg L^− 1^, time (15, 30, 45, 60, 90, 120 min), stirring 100 rpm, concentration 50 mg L^− 1^].

Concurring to Table 2, the PSO model fits the data better than other kinetic isotherm models, with a lower error sum (2.137), as illustrated in Fig. 15.

**Table 2:** Parameters for different kinetic isotherm models.

	Isotherm model	Error sum
		RYS3R
1.	Avrami	5.273
2.	Pseudo-first-order	6.794
3.	Pseudo-second-order	2.137
4.	Elovich	8.579
5.	Intra-particle diffusion	5.913

### Thermodynamic study

**Fig. 16:**
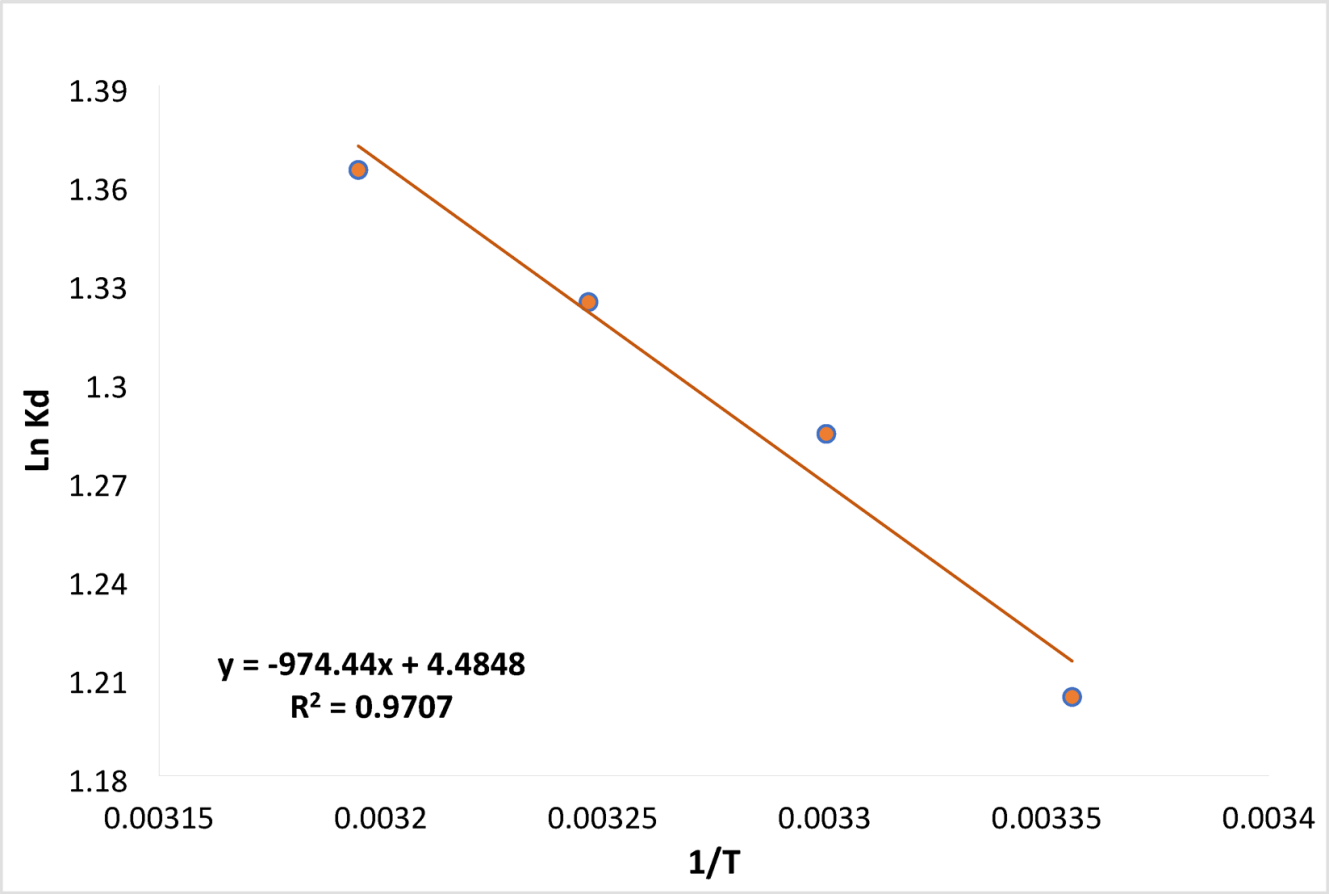
Thermodynamic linear study for the removal of RYS3R [pH 6, dose 0.2 mg L^− 1^, time 60 min, temp. (298, 303, 308, 313 K), stirring 100 rpm, concentration 50 mg L^− 1^].

Figure 16 illustrates the thermodynamic parameters calculated for the different adsorption processes and their impact on the rates. As seen, all the values for the change in Gibbs free energy ∆G (− 2.983, − 3.235, − 3.391, and − 3.551 kJ mol^−1^) were negative at various temperatures of 298, 303, 308, and 313 K, providing evidence that Ficus-nZVCu materials may spontaneously react with RYS3R dyes, respectively. The absolute value of ∆G increased as the temperature increased, indicating that the reaction was becoming more spontaneous and that high temperatures were favorable for the reaction, which points to a chemisorption mechanism. For RYS3R dyes, the enthalpy change was positive (8.065), indicating an endothermic reaction. As a result, the rate of RYS3R dye reduction at equilibrium increases as the temperature rises. In the system, the entropy change ∆S was also greater than zero (0.037), indicating that entropy increased during the adsorption process. When RYS3R dyes were exchanged on the surface of Ficus-nZVCu materials, the entropy increased because solid-liquid interactions during the adsorption process made the system more random^45,46^.

### Statistical analysis

Utilizing the proposed technique, the impact of variables on the removal processes of RYS3R dyes was investigated. It was found that R^2^ = 0.931, the standard error of the estimate is 7.119, the study’s error rate is low, and the examined factors account for nearly 93% of all the variables influencing the removal process, respectively. The **ANOVA** program indicated that the model is significant, as evidenced by a* P*-value of less than 0.05.

#### Response surface methodologies

Utilizing linear regression analysis (IBM-SPSS Statistics), the impact of various studied parameters on the adsorption processes of dyes was investigated, and the results corroborated the practical findings. The information obtained demonstrated that all factors impacted the removal process, except for stirring rate, and temperature, which can be disregarded because their P values are greater than 0.05, as shown in Table 3. The B values are used for RYS3R in Eq. 8 to figure out the removal, while:

8 $${\text{R}}\% = 125.589 + \left( { - 15.295} \right){\text{X1}} + \left( {0.431} \right){\text{X2}} + \left( {229.699} \right){\text{X3}} + \left( { - 0.039} \right){\text{X4}} + \left( { - 0.748} \right){\text{X5}} + \left( { - 0.351} \right){\text{X6}}$$

**Table 3:** Statistical analysis.

Model	RYS3R	
	B	Sig
[Constant]	125.589	0.012
pH	−15.295	0.006
Time	0.431	0.029
Dose	229.699	0.000
Stirring	−0.039	0.498
Conc	−0.748	0.000
Temp	−0.351	0.665

#### Artificial neural network

The multilayer perceptron neural network model 6-1-3 was used to train the ANN model for RYS3R dye removal, utilizing both sample training and testing without omitting any findings. Table 4 shows the sum of squared errors for testing and training, as well as the relative errors, for a total of 26 runs.

**Table 4:** Model summary.

Model	RYS3R	
	Training	Testing
The sum of squares error	0.073	0.209
Relative error	0.008	0.299

**Fig. 17:**
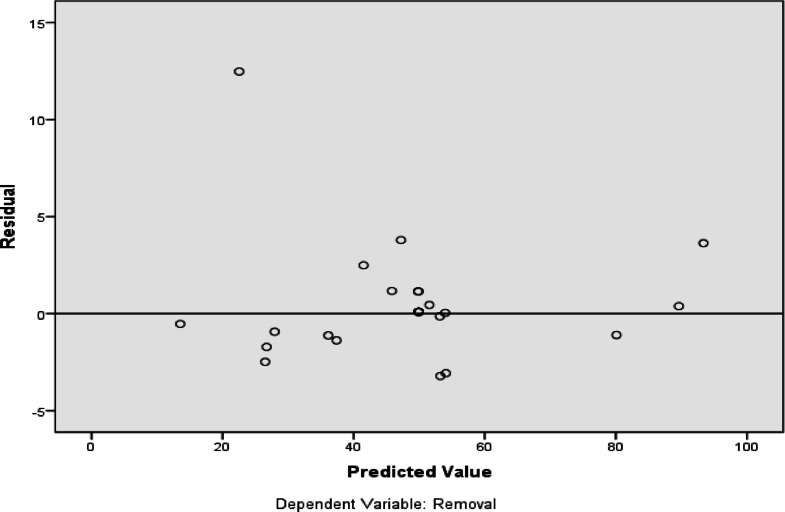
RYS3R dye residual values.

**Fig. 18:**
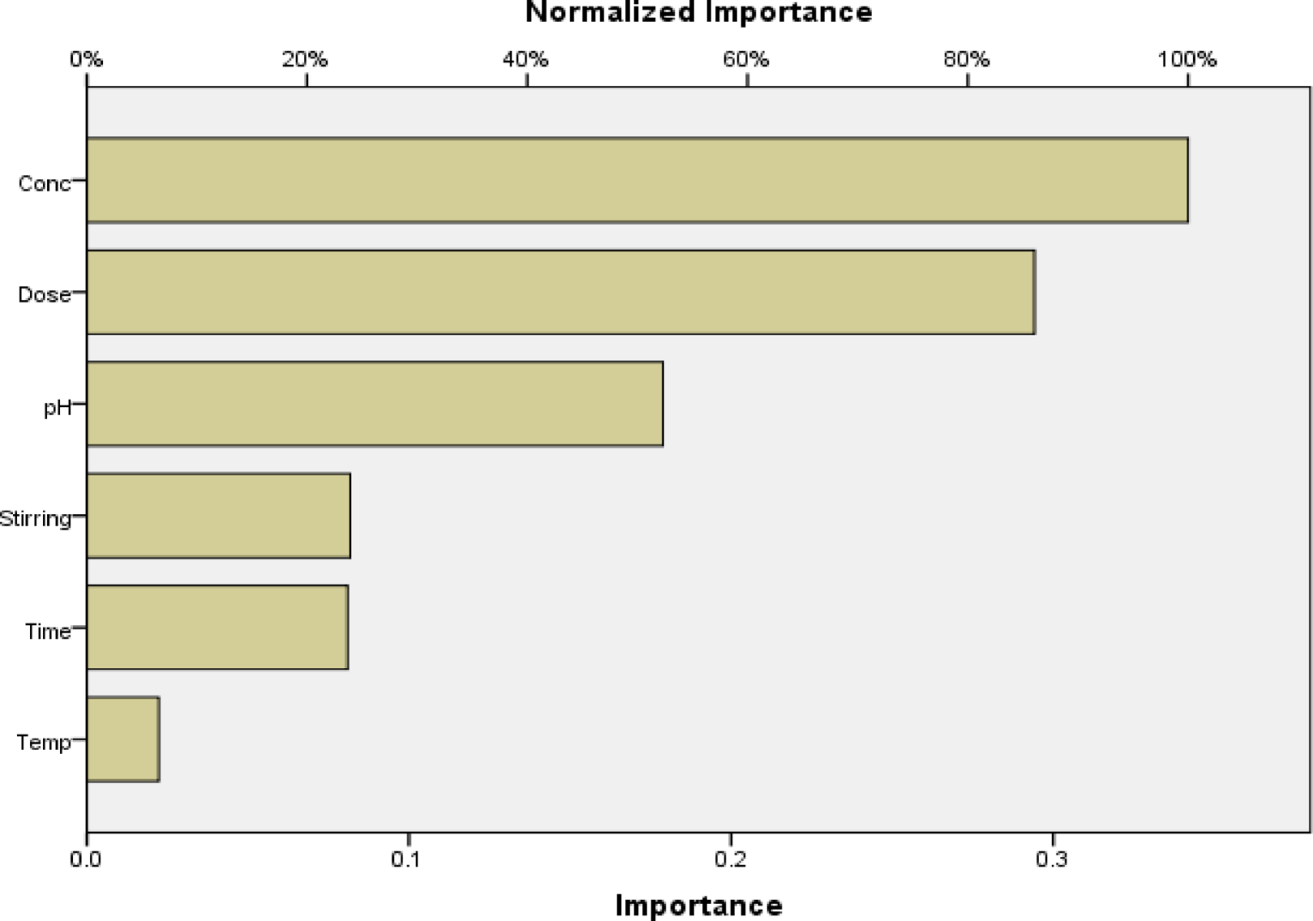
RYS3R importance for each co-variable.

Figures 17 demonstrate that there is a negligible difference between the residual value and the predicted value, showing the accuracy of the model’s predictions for the adsorption of RYS3R dye onto the Ficus-nZVCu surface. Figure 18 illustrates the significance of each co-variable in the removal efficiency, with a dose for RYS3R being the most useful parameter. The stirring rate for RYS3R is the least useful. The ANN results agreed with the RSM findings and the experimental data.

### Reusability of FB-ZVI NPs

**Fig. 19:**
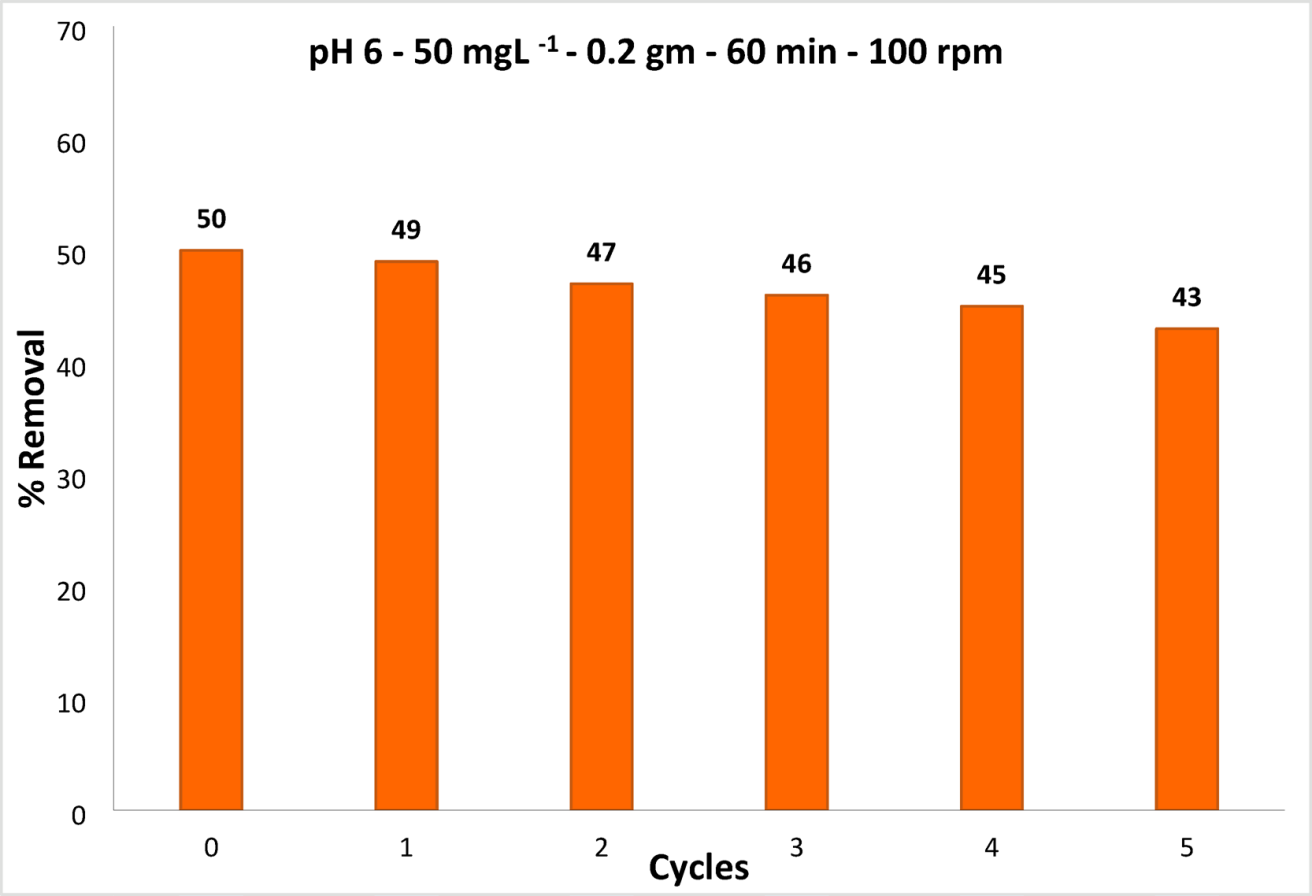
Ficus -ZVCu particles reuse.

Figure 19 implies that the removal efficiencies decreased with each reuse run of RYS3R. Using 0.2 g L^− 1^ of Ficus-nZVCu at solution pH 6 and a stirring rate of 100 rpm, the percentage of dye removal for RYS3R dye concentrations of 50 mg L^− 1^ was determined after five recycles. However, the removal efficiency of RYS3R was still high. Differing in the appropriateness of removal, this might have been caused by the loss of nano-absorbent or by the irreversible filling of adsorption sites. The regenerated adsorbent retained good adsorption capability after the 5th round of recycling. Results show that Ficus-nZVCu proposals have a high potential for repeated use in removing these dyes without a significant decrease in removal suitability.

Finally, based on the observed results and the productive discussion, we can confidently declare that Ficus-nZVCu is an environmentally friendly adsorbent that effectively removes azo dyes RYS3R from textile wastewater, creating high-quality treated effluent.

## Conclusion

The green synthesis of eco-friendly Ficus-ZVCu nanoparticles was successful using low-cost Ficus Benjamina leaves. SEM and FT-IR were utilized to confirm the production of porous nanoparticles. Under optimum conditions, the Ficus-nZVCu particles are efficient nano-adsorptive agents for removing RYS3R from wastewater. The optimum removal efficiency of RYS3R dye is 50% with the following parameters: dose 0.2 g L^**− 1**^, time 60 min, concentration 50 g L^**− 1**^, and solution pH 6. Langmuir and pseudo-second-order models are more fitting for both linear and non-linear adsorption models. The adsorption thermodynamic parameters showed the nature of spontaneity adsorption, chemisorption, endothermic processes, and random adsorption interference, which increased at the solid-solution interface and had a reasonable adsorption affinity of RYS3R dye molecules towards nanoparticles. The ANOVA software results revealed that nearly 94% of the variables influencing the RYS3R dye removal processes, respectively. The regenerated adsorbent still retained fit adsorption capability after the fifth round of recycles.

## Supplementary Information

Below is the link to the electronic supplementary material.

Supplementary Material 1

## Data Availability

The data that support the findings of this study are available from the corresponding author upon reasonable request.
