# Supplementary material for: Adsorptive removal of reactive yellow S3R dye from aqueous solutions using green-synthesized copper nanoparticles
Source: Sci Rep. 2026 Jan 6;16:800. doi: 10.1038/s41598-025-32372-5 (PMC12780057; doi:10.1038/s41598-025-32372-5)
Supplement: Supplementary file 1 — Supplementary Material 1 [file 41598_2025_32372_MOESM1_ESM.docx]

**Adsorption equilibrium models**

**Table S1.** Parameters for different adsorption isotherm models

|  | **Isotherm model** | **Linear equations** | **Parameters** |
| --- | --- | --- | --- |
|  | Dubinin-Radushkevich | L𝑛 q_e_= L𝑛 q_m_ - (β * E^2^) | ***Β*** Dubinin-Radushkevich isotherm constant  ***Ε*** Polanyi potential (J/mol) |
|  | Elovich | L𝑛 q_e_/C_e_= L𝑛 K * q_m_ – 1/q_m_ * q_e_ | ***q_m_*** capacity of the adsorbent monolayer (mg/g) |
|  | Flory–Huggins | L𝑛 𝜃/𝐶_𝑒_ = L𝑛 𝐾 + 𝑛 * L𝑛 (1−𝜃) | **𝜃** degree of surface coverage  ***K*** Flory–Huggins model equilibrium constant  ***N*** number of adsorbates occupying adsorption site |
|  | Fowler | L𝑛 [𝐶_𝑒_(1−𝜃)/𝜃] = − L𝑛 𝐾 + (2𝑊/𝑅𝑇) * 𝜃 | ***K*** Fowler constant  ***W*** empirical interaction energy between two adsorbed molecules at the adjacent neighboring site (kJ/mol)  **𝜃** fractional coverage of the surface |
|  | Freundlich | L𝑛 q_e_ = L𝑛 K+ n * L𝑛 C_e_ | ***K*** Freundlich constant  **𝑛** value indicating the degree of linearity between the adsorbate solution and the adsorption process |
|  | Halsey | q_𝑒_ =(1/𝑛) * L𝑛 𝐾− (1/𝑛) * L𝑛 𝐶_𝑒_ | ***K*** and ***n*** are the Halsey model constants |
|  | Harkins-Jura | 1/𝑞_𝑒_ ^2^ = (𝛽/𝐴) − (1/𝐴) * L𝑜𝑔 𝐶_𝑒_ | **𝛽** value is related to specific surface area of adsorbent  **𝐴** are the Harkin Jura isotherm constants |
|  | Henry | q_e_ = K * C_e_ | ***q_e_*** amount of adsorbed adsorbate molecule per gram of adsorbent (mg/g) |
|  | Hill-Deboer | L𝑛 [𝐶_𝑒_(1−𝜃)/𝜃] −(𝜃/1−𝜃) = −L𝑛 𝐾− (𝐾_2_/𝑅𝑇) * 𝜃 | ***K*** Hill-Deboer constant (L/mg)  ***K_2_*** is the energetic constant of the interactions |
|  | Jovanovic | L𝑛 q_𝑒_ = L𝑛 q_𝑚_ − 𝐾 * 𝐶_𝑒_ | ***q_e_*** is the amount of adsorbate in the adsorbent at equilibrium (mg/g)  ***q_m_*** maximum uptake of adsorbate  ***K*** Jovanovic constant |
|  | Langmuir | C_e_/q_e_ = 1/(q_𝑚_ * 𝐾) +(1/q_m_)* C_e_ | ***Ce* is** the adsorbate equilibrium concentration (mg/L)  ***K*** is the Langmuir adsorption constant. |
|  | Redlich-Peterson | L𝑛 (C_e_/q_e_) = − L𝑛 A + B * L𝑛 C_e_ | ***q_e_*** amount of adsorbed adsorbate molecule per gram of adsorbent (mg/g) |
|  | Sips | L𝑛 (q_e_/(q_m_−q_e_) = L𝑛 (K)1/n + (1/n) * L𝑛 C_e_ | ***q_m_*** capacity of the adsorbent monolayer (mg/g) |
|  | Temkin | q_e_ = B * L𝑛 A + B * L𝑛 C_e_ | ***B*** adsorption heat constant (KJ/mol)  ***A*** binding equilibrium constant  ***T*** absolute temperature |
|  | Toth | L𝑛 (q_e_/q_m_-q_e_)= n * L𝑛 K + n * L𝑛 C_e_ | ***q_e_*** amount of adsorbed adsorbate molecule per gram of adsorbent (mg/g) |

**Kinetic equilibrium models**

**Table S2.** Parameters for different kinetic isotherm models

|  | **Isotherm model** | **Linear equations** | **Parameters** |
| --- | --- | --- | --- |
|  | Pseudo first order | L𝑛 (q_e_- q_t_) = L𝑛 q_e_ – K_1_ * t | **q_e_**, **q_t_** are the sorption capacity equilibrium (mg/g)  **k_1_** rate constant (min^-1^) |
|  | Pseudo second order | t/q_t_ = 1/q_m_ * q_e_^2^ + 1/q_e_ * t | **q_e,_** **q_t_** are the sorption capacity equilibrium (mg/g) |
|  | Elovich | q_t_ =1/α L𝑛 α B + 1/ α * L𝑛 t | ***B*** Elovich model constant  ***α*** the initial sorption rate (mg/g min) |
|  | Intra-particle diffusion | q_t_ = 𝐾_diff_ * t ^½^ + C | ***k_diff_*** intra-particle diffusion rate constant (mg/ (g min^1/2^))  ***C*** a constant related to the thickness of the boundary lay (mg/g) |
|  | Avrami | L𝑛 [L𝑛 (q_e_/q_e_−q_t_)] =  n L𝑛 𝐾 + n L𝑛 t | ***K*** Avrami constant  ***𝑛*** value indicating the degree of linearity between the adsorbate solution and the adsorption process |
|  | Bangham | Log (C_0_/C_0_−mq_t_) = Log (m*K/2.303V) + ΔB Log t | ***C_0_*** the initial concentration of adsorbate in solution (mg/L)  ***V*** the volume of solution (mL)  ***m*** is the weight of adsorbent per liter of solution (g/L)  ***α, k*** are constants |
|  | Boyd | B_t_ = K * t  B_t =_ − 0.4977 − L𝑛 (1-(q_t_/q_e_)) | ***K*** Boyd constant |
|  | liquid film diffusion | L𝑛 (1− q_t_/q_e_) = − 𝐾 * t | ***K*** constant |

**Adsorption isotherm investigations**

**Table S3. Linear adsorption isotherm models:**

|  | **Isotherm model** | **RRS3B** |
| --- | --- | --- |
|  | **Dubinin-Radushkevich** | *K*= 2.00*10^-7^  *q*_m_= 130.490  *R* ^2^= 0.8503 |
|  | **Elovich** | *K*= 0.9996  *q*_m_= 137.94  *R* ^2^= 0.9805 |
|  | **Flory–Huggins** | *K*= 0.9842  *n*= - 0.005  *R* ^2^= 0.946 |
|  | **Fowler** | *K*= 2.077*10^-10^  *W*= -2.218  *R* ^2^= 0.9934 |
|  | **Freundlich** | *K*= 106.762  *n*= 18.904  *R* ^2^= 0.9879 |
|  | **Halsey** | *K*= 7.969*10^-12^  *n* = -0.154  *R* ^2^= 0.9822 |
|  | **Harkins-Jura** | *A*= 17.361  *B*= 2.078  *R* ^2^= 0.9832 |
|  | **Henry** | *K*= 0.312  *q*_m_= 114.3  *R* ^2^= 0.9232 |
|  | **Hill-Deboer** | *K*_1_= 4.817*10^-8^  *K*_2_= -3.384  *R* ^2^= 0.9736 |
|  | **Jovanovic** | *K*= -0.0025  *q*_m_= 114.366  *R* ^2^= 0.910 |
|  | **Langmuir** | *K*= 0.745  *q*_m_= 136.986  *R* ^2^= 0.9992 |
|  | **Redlich-Peterson** | *A*= 110.454  *B*= 0.9684  *R* ^2^= 0.9989 |
|  | **Sips** | *K*= 5.369  *n*= 1.699  *R* ^2^= 0.8880 |
|  | **Temkin** | *A*= 1.255  *B*= 6.484  *R* ^2^= 0.9822 |
|  | **Troth** | *K*= 5.369  *n*= 0.5887  *R* ^2^= 0.888 |

**Figure S1. Isotherm studies for linear adsorption models**

**Kinetics studies**

**Table S4. Parameters for different kinetic isotherm models**

|  | **Isotherm model** | **RRS3B** |
| --- | --- | --- |
|  | **Pseudo-first order** | *K*_1_= 0.06  *q*_e_= 66.023  *R* ^2^= 0.960 |
|  | **Pseudo-second order** | *K*_2_= 0.0016  *q*_e_= 90.909  *R* ^2^= 0.9993 |
|  | **Elovich** | *α*= 0.0814  *B*= 139.10  *R* **^2^**= 0.9169 |
|  | **Intra-particle diffusion** | *C*= 52.682  *K*= 3.3655  *R* **^2^**= 0.8112 |
|  | **Avrami** | *K*= 0.082  *n*= 0.7972  *R* **^2^**= 0.9670 |
|  | **Bangham** | Δ*B*= 0.2284  *K*= 1.2813  *R* **^2^**= 0.9058 |
|  | **Boyd** | *K*= 0.0579  C= -0.247  *R* **^2^**= 0.9617 |
|  | **Liquid film diffusion** | *K*= 0.0579  C= -0.251  *R* **^2^**= 0.9617 |

**Figure S2. Kinetic studies for linear models**
